# Diagnostic Caveats and Clinical Pitfalls in the Overlap of Leprosy and Para-Kala-Azar Dermal Leishmaniasis

**DOI:** 10.4269/ajtmh.26-0451

**Published:** 2026-08-18

**Authors:** Rishikesh Kumar, Devendra Prasad Yadav, Sanjay Kumar Chaturvedi, Krishna Pandey

**Affiliations:** ICMR Rajendra Memorial National Institute of Health Research-Agamkuan Patna Bihar India E-mails: drkumarrishikesh@gmail.com; devendrarmrims@gmail.com; sanjayrmri@rediffmail.com; drkrishnapandey@yahoo.com

Dear Editor,

We read with interest the Images in Clinical Tropical Medicine report by Kwan et al.^1^ regarding a 35-year-old Bangladeshi man diagnosed with concurrent visceral leishmaniasis (VL) and post-kala-azar dermal leishmaniasis (PKDL)—termed para-KDL—following initial misdiagnosis and management for leprosy. While this case underscores the epidemiological relevance of imported leishmaniasis in non-endemic settings and the post-elimination landscape in Bangladesh,^2^ several technical, clinical, and diagnostic gaps in the report warrant rigorous scrutiny.

First, the primary clinical pitfall stems from the baseline evaluation for leprosy. The authors note that the patient presented with infiltrated plaques and patches but omit crucial sensory and neurological assessments essential for differentiating leprosy from PKDL.^3^ Classic hallmarks of leprosy, such as asymmetric lesion distribution, localized sensory loss, ulnar or radial nerve enlargement, clawed hand, or foot drop, are unaddressed.^3^

Furthermore, the initial therapeutic choice raises concerns. Prednisolone is indicated for Type 1 lepra reactions;^4^ however, the patient did not present with an active reactional state at baseline. The administration of a potent systemic corticosteroid appears medically unjustified and likely masked underlying pathology while precipitating systemic visceralization of leishmaniasis. To scientifically validate the authors’ hypothesis regarding steroid-induced flares, clinical images documenting reactional skin flares post-prednisolone tapering should have been provided.

Second, the laboratory workup presents quality control and technical concerns. The authors attribute the initial slit-skin smear (SSS) positivity for acid-fast bacilli to “staining artifacts or cross contamination”. Dismissing a positive SSS as a contaminant without presenting the underlying Ziehl-Neelsen or Fite-stained microscopic images for technical vetting weakens the diagnostic reliability of the report.

**Figure 1.**
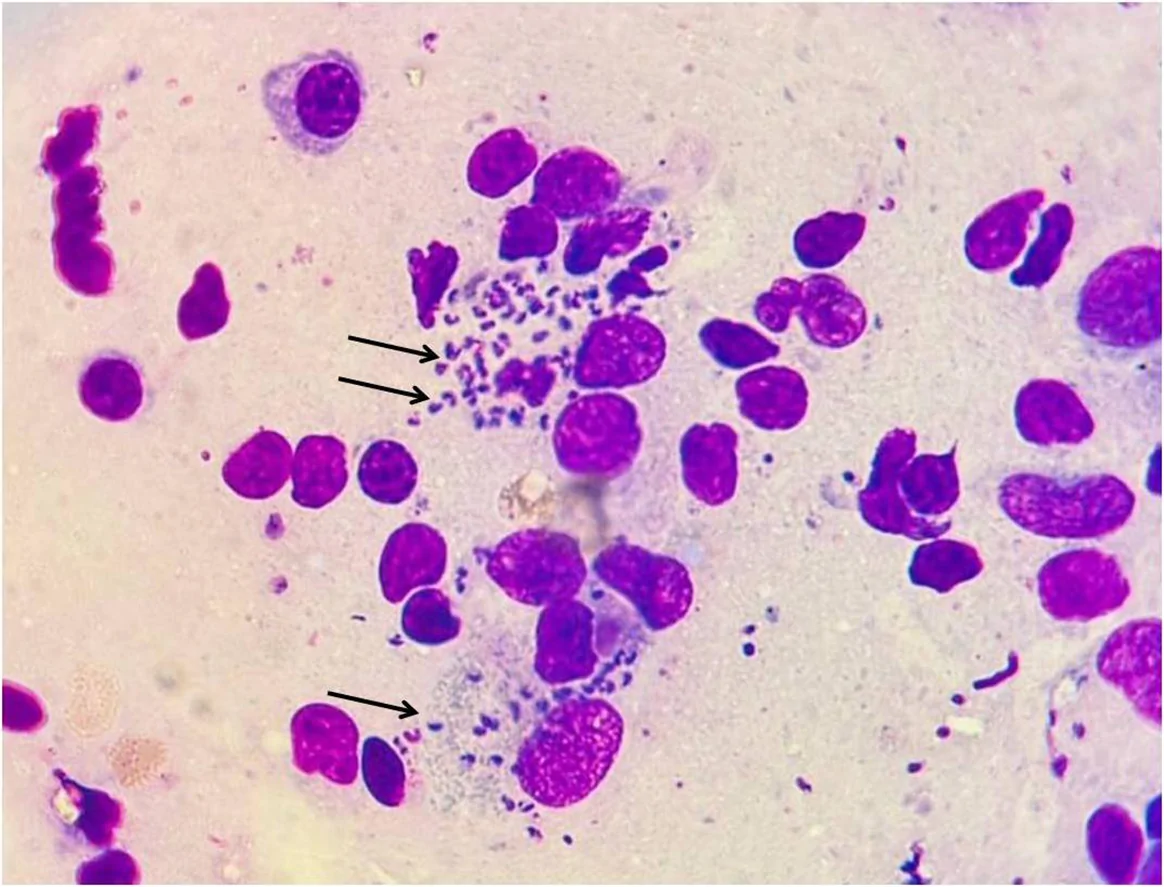
Giemsa-stained slit skin smear containing Leishmania Amastigotes indicated by black arrows (oil immersion, 1000× magnification).

Additionally, the histological images provided suffer from poor magnification and structural clarity, making the definitive identification of Leishman-Donovan (LD) bodies highly speculative. Standard Giemsa staining typically imparts a distinct purple hue to the kinetoplast and nucleus of amastigotes (see Figure 1 example provided here); this coloration is unconvincing in Figure 2 of the published report. For a definitive diagnosis of VL/PKDL, high-magnification confirmation or molecular corroboration via nucleic acid amplification tests should have been pursued.

Finally, the clinical evaluation of systemic visceralization lacks completeness. While the patient exhibited hepatosplenomegaly, other classic hallmarks of VL in the Indian subcontinent, such as progressive weight loss, severe anemia, gastrointestinal disturbances, and the characteristic darkening of the skin, were not reported. Given that the patient experienced blurred vision during flares, an objective ophthalmological evaluation would be helpful to ascertain whether this symptom stemmed from an underlying pathology or represented an adverse manifestation of corticosteroid use.

In conclusion, while this case highlights a rare and complex presentation of para-KDL, it underscores the danger of initiating empirical immunosuppressive therapy without robust, multi-parametric diagnostic confirmation. In an era in which global migration blurs endemic boundaries, ensuring strict technical quality control in diagnostic microscopy is paramount to prevent clinical missteps.
